# Demonstration of Fully Integrable Long-Range Microposition Detection with Wafer-Level Embedded Micromagnets

**DOI:** 10.3390/mi13020235

**Published:** 2022-01-30

**Authors:** Björn Gojdka, Daniel Cichon, Yannik Lembrecht, Mani Teja Bodduluri, Thomas Lisec, Markus Stahl-Offergeld, Hans-Peter Hohe, Florian Niekiel

**Affiliations:** 1 Fraunhofer Institute for Silicon Technology ISIT, Fraunhoferstr. 1, 25524 Itzehoe, Germany; yannik.lembrecht@isit.fraunhofer.de (Y.L.); mani.teja.bodduluri@isit.fraunhofer.de (M.T.B.); thomas.lisec@isit.fraunhofer.de (T.L.); florian.niekiel@isit.fraunhofer.de (F.N.); 2 Fraunhofer Institute for Integrated Circuits IIS, Am Wolfsmantel 33, 91058 Erlangen, Germany; markus.stahl-offergeld@iis.fraunhofer.de (M.S.-O.); hans-peter.hohe@iis.fraunhofer.de (H.-P.H.)

**Keywords:** integrated position detection, micro positioning, MEMS, Hall sensor, micromagnets

## Abstract

A fully integrable magnetic microposition detection for miniaturized systems like MEMS devices is demonstrated. Whereas current magnetic solutions are based on the use of hybrid mounted magnets, here a combination of Hall sensors with a novel kind of wafer-level integrable micromagnet is presented. 1D measurements achieve a precision <10 µm within a distance of 1000 µm. Three-dimensional (3D) measurements demonstrate the resolution of complex trajectories in a millimeter-sized space with precision better than 50 µm in real time. The demonstrated combination of a CMOS Hall sensor and wafer-level embedded micromagnets enables a fully integrable magnetic position detection for microdevices such as scanners, switches, valves and flow regulators, endoscopes or tactile sensors.

## 1. Introduction

Various microsystem applications require the precise monitoring of the position of a moving microstructure, for example MEMS scanners [1], tactile sensors [2,3] or actuators [4]. For this purpose, mainly capacitive [5] and piezoresistive [6] sensor elements are currently used as integrated solutions. An essential disadvantage of capacitive and piezoresistive position detection is that signal acquisition requires electrical and mechanical connections to the sensory elements. Accordingly, the measurement cannot be contact-free and requires special elastic structures within the MEMS device. The larger the travel distance, the larger the elastic structures must be to allow for the corresponding deformation. In addition, mechanical coupling with the actuator occurs in both cases. In the case of piezoresistive position detection, additional force is required to deform the piezoresistive elements. In the case of capacitive position detection, the force to be overcome results from the electrostatic interaction. An alternative is magnetic position detection, based on the observation of the magnetic field of a permanent magnet attached to the moving structure. In this case, position detection takes place in a contactless way and without force over comparatively long distances.

In general, a basic magnetic position detection scheme is based on monitoring changes of a magnetic field created by the displacement of permanent magnets. For a miniaturized magnetic position detection system at least one permanent micromagnet is rigidly mounted to the movable microstructure, such that both undergo an identical translation during movement. A magnetic field sensor is rigidly mounted to a base part of the microsystem which represents the reference point, e.g., [7]. A translation of the permanent magnet in the movable structure results in a change in magnetic flux density measured by the sensor. The changes in measured magnetic flux density can be utilized to derive the translation of the permanent magnet and thus determine the position of the movable structure [8,9].

**Figure 1:**
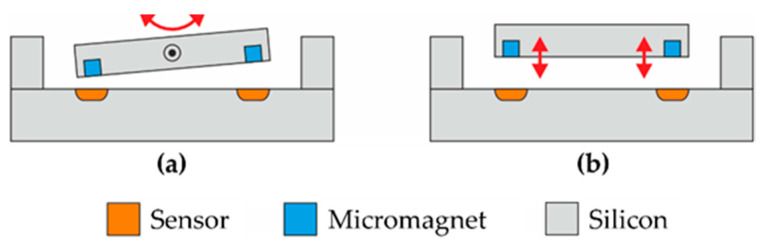
Schematic illustrations of integrated position detection systems based on arrangements of Hall sensors and micromagnets. The rotating device shown in (**a**) represents microelectromechanical systems (MEMS) mirror scanners or joysticks, among others. The translational arrangement in (**b**) could be used for an autofocus device, for example. One possibility for manufacturing the depicted assemblies in silicon technology is wafer bonding [13].

Mass producibility is a basic requirement for relevant technical applicability. So far, only discrete magnets have been available for magnetic position detection in microsystems. The magnets must be integrated hybrid into the system [9,10], thus complicating the fabrication process accompanied with lower precision and higher costs. Using a powder-based microfabrication process [11] micromagnets can now be precisely integrated back-end-of-line (BEOL) compatible on wafer level [12]. This integration technology opens up the prospect to realize a fully integrated magnetic position detection in MEMS. Examples of such integrated arrangements are illustrated in Figure 1. In the following study, precise long-range magnetic 3D microposition detection is demonstrated by combining CMOS-fabricated 3D Hall sensors with wafer-level embedded NdFeB micromagnets.

## 2. Theory

**Figure 2:**
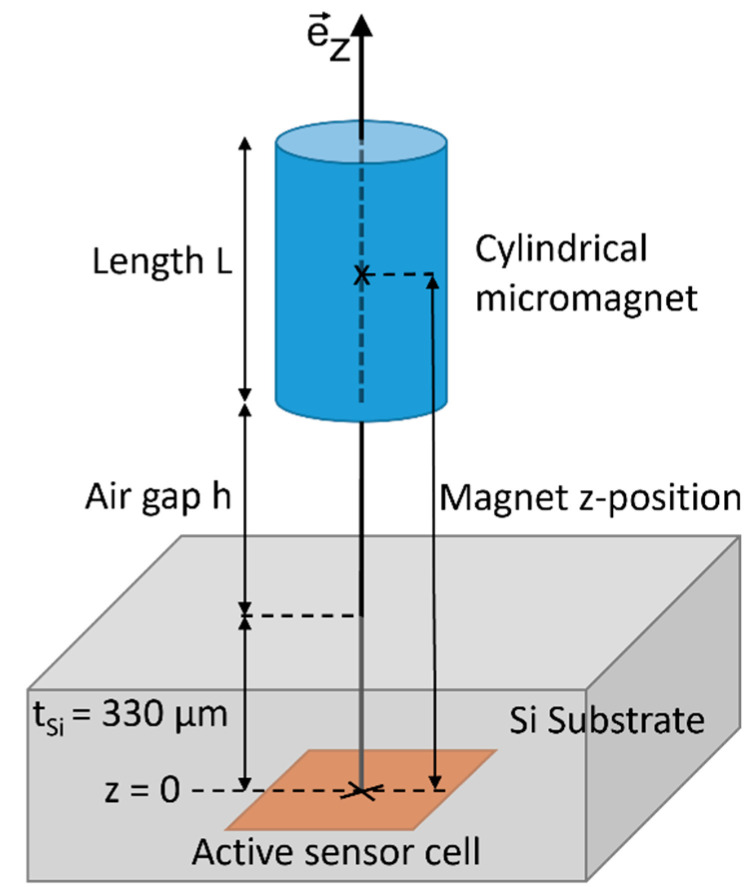
Schematic illustration of the proposed position sensing setup introducing the coordinate system used throughout this work. The position of the magnet z is defined by the distance between the active sensor cell and the center of the magnet at L/2. The thickness of the silicon substrate is t_Si_ = 330 µm in the case of the unpackaged Hall sensor IC FH3D02 of Fraunhofer IIS.

Figure 2 schematically illustrates the geometry referred to throughout this work. The arrangement is based on a concentric parallel configuration: a permanent cylindrical magnet is positioned relative to a sensor along the same axis (z-direction) as its magnetization and the translational movement Δz. In the following, the origin of the coordinate system z = 0 is assigned to the center of the sensor cell, while the z-position of the magnet is referenced with respect to the center of the magnet at L/2 within the cylinder. The calculations are performed for a magnet with L = 590 µm since the same length is used in the following experiments. Note that in this case the magnet is in contact with the sensor cell at the position z = L/2 = 295 µm (neglecting the presence of the silicon substrate in the theoretical calculations).

**Figure 3:**
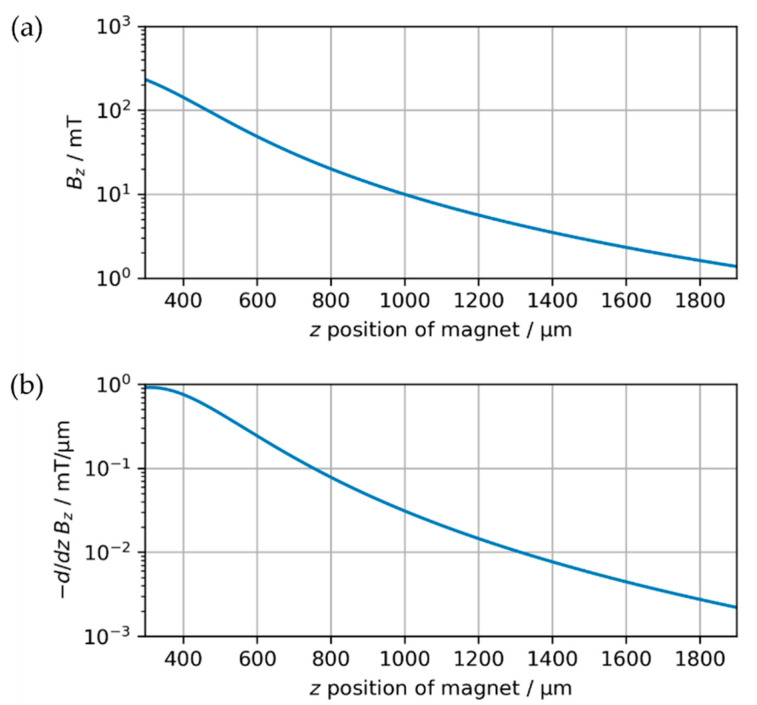
Theoretical calculation of the magnetic field with respect to the position z of a cylindrical magnet with diameter d = 500 µm, length L = 590 µm and magnetization M = 500 mT/µ_0_. Note that z = 295 µm (= L/2) corresponds to contact between the magnet and the active sensor cell according to Figure 2, neglecting the presence of the Si substrate in this theoretical analysis. (**a**) Magnetic flux density in z-direction B_z_. (**b**) Gradient of magnetic flux direction in z-direction.

Theoretical calculations have been executed numerically integrating the Biot–Savart law over the surface current due to magnetization M:

(1) $$\vec{B}\left(\vec{r}\right)=\vec{\nabla }\;\times \frac{{I\;µ}_{0}}{4\;\pi }\;\int \frac{d\vec{l}}{r}$$

with total current I = M∙L and $d\vec{l}$ representing an infinitesimal part of the surface pointing in current direction. Figure 3a shows the magnetic flux density B_z_ at the sensor with respect to the position of the cylindrical magnet (diameter d = 500 µm, length L = 590 µm, magnetization M = 500 mT/µ_0_). The calculations confirm that the method is suitable for long distances, as even at separations of more than 1600 µm the magnetic flux density is still above 1 mT. The derivative of B_z_ with respect to z shown in Figure 3b yields the gradient of the flux density at the sensor. B_z_(z) is strongly non-linear implying the necessity of implementing a reference function to convert the magnetic flux density into a position. Furthermore, the flux density gradient decreases with increasing distance. Accordingly, a higher sensor accuracy enables the determination of positions at greater distances. As shown in Figure 3b, the use of a sensor capable of resolving differences in magnetic flux density down to 0.01 mT would allow the position of the exemplary magnet to be monitored with an accuracy of up to 1 µm to distances greater than z = 1250 µm. Besides the specifications of the utilized sensor, this performance can be tuned by shaping the magnetic field with customized geometries or arrays of integrated permanent magnets.

## 3. Experimental Setup

A fully integrable micromagnetic detection is demonstrated for one and three degrees of freedom using a combination of wafer-level integrable micromagnets and CMOS Hall sensors. The corresponding components and setups are described below.

### 3.1. Powder-Based Wafer-Level Integrated Micromagnets

**Figure 4:**
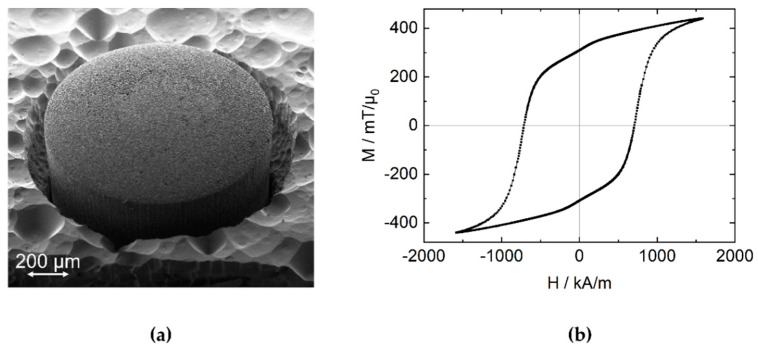
(**a**) Scanning electron microscopy (SEM) micrograph of an embedded micromagnet with a diameter d = 1000 µm and L = 590 µm made of NdFeB micropowder which was solidified with an Al_2_O_3_ layer deposited by atomic layer deposition (ALD). The surface roughness of the magnet results from the powder-based manufacturing process. The silicon substrate was partially removed by XeF_2_ etching for illustration. The rough surface of the remaining substrate is typical for XeF_2_ etching. (**b**) Ferromagnetic hysteresis from the magnet in (**a**) measured by VSM. The magnet exhibits an intrinsic coercivity of H_c_ = 707 kA/m and a remanent magnetization of M_r_ = 310 mT/µ_0_.

The precise BEOL compatible integration of three-dimensional magnetic microstructures into microsystems presents a challenge on the substrate level. A novel technique [11,14] based on microfine powder solidified by atomic layer deposition (ALD) is facilitated to create magnetic volumes on the substrate level [12]: cylindrical microcavities of various diameters were etched to a target depth of 600 µm by deep reactive ion etching (DRIE) into 8-inch Si wafers. Subsequently, NdFeB powder (Magnequench MQFP-B+, D50 = 5 µm) was dry-filled [15] into the micromolds. Depending on the diameter and position on the wafer, the DRIE process yields varying actual cavity depths with a standard deviation of about 15 µm across the wafer. The powder was solidified with a 75 nm thick Al_2_O_3_ layer deposited by ALD at 75 °C using the precursors trimethylaluminum (TMA) and water. Figure 4a displays a magnet with d = 1000 µm which is partially released from the substrate by XeF_2_ etching for illustration. The magnets were magnetized along the cylinder axis either individually at H = 1600 kA/m using a vibrating sample magnetometer (VSM 7400, Lake Shore Cryotronics, Westerville, United States ) or on wafer-level in a custom magnetization tool (MAGSYS, Dortmund, Germany) at 2800 kA/m. The characterization by VSM yields an intrinsic coercivity of H_c_ = 707 kA/m and typical remanent magnetization of M_r_ = 250–450 mT/µ_0_ as displayed in Figure 4b. For the following experiments, magnets with d = 500 µm and L = 580–590 µm were used to demonstrate the potential miniaturization. The magnets were released from the substrate by DRIE, leaving a 150 µm-thick silicon frame around each magnet for further handling.

### 3.2. Setup of One-Dimensional (1D) Measurement

**Figure 5:**
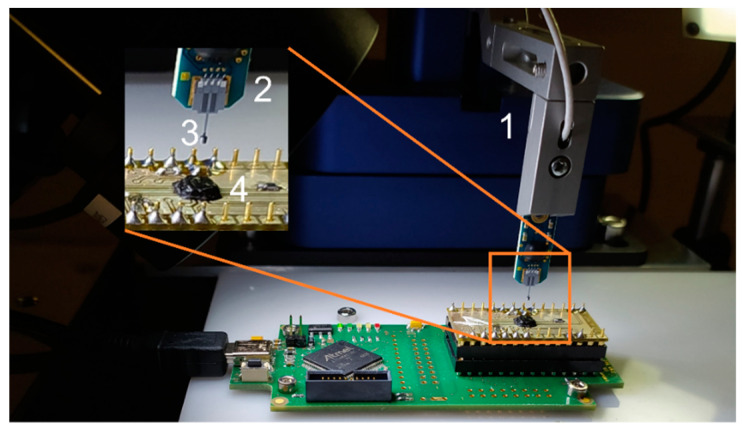
Setup for 1D measurements: the micromanipulator (1) positions a force probe (2) with an attached micromagnet (3) relative to a Hall sensor (4).

A microrobotic system (FemtoTools FT-RS1002) is used for precise positioning of the micromagnets with respect to a 3D Hall sensor (Fraunhofer IIS FH3D02 [16]) as depicted in Figure 5. The magnets are glued to the tips of cantilever-like microforce sensing probes (FemtoTools FT-S100000). Prior to the z-axis measurement, the magnet is brought into a defined initial position with respect to the Hall sensor; first, the magnet is positioned so that the Hall sensor outputs B_x_ and B_y_ are minimized to bring the z-symmetry axis of the B-field in line with the sensor cell. Then, the magnet is brought into mechanical contact with the Hall sensor, indicated by an increase in the force sensed by the probe during the approach in the z-direction. This point of contact is defined as h = 0, with h being the air gap between sensor substrate surface and magnet. Subsequently, while the probe is retracted B_z_(h) is measured with the Hall sensor (B_z_) and the micromanipulator (h). Note that although a 3D Hall sensor is used, a 1D Hall sensor would suffice for 1D position detection integrated into a MEMS device. In this case, the alignment of the magnet to the sensor is accomplished during the manufacturing process.

### 3.3. Setup of Three-Dimensional (3D) Measurement

**Figure 6:**
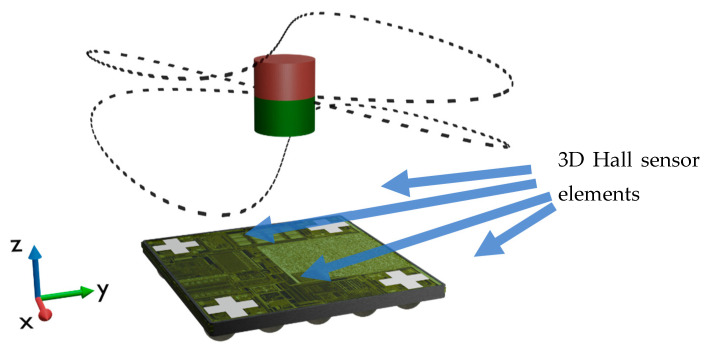
Setup for 3D measurements, consisting of a Hall sensor IC with 4 measurement cells and the cylindrical magnet. The trajectory realized with a 6D manipulator is indicated by the dashed curve (see Supplement S1 for animation).

To determine the position of the magnet in three dimensions all four measurement cells of a FH3D04 IC (Fraunhofer IIS) are used. Accordingly, the four Hall sensors of the IC deliver the magnetic field vectors at four different positions with a pitch of 1.5 mm as illustrated in Figure 6.

A 6D manipulator (hexapod) moves the magnet (d = 500 µm, L = 580 µm) along a trajectory constructed from sine curves with amplitudes of 1.5 mm for the x and y components and 0.5 mm for the z component (refer to Supplement Animation S1). The surface of the magnet remains parallel to the chip surface throughout the motion. During the movement of the magnet, a microcontroller (CORTEX-M4F) processes the magnetic flux density values measured by the four 3D Hall sensor elements to estimate the position of the magnet. The post-processing uses an unscented Kalman filter [8] and the quasi-analytical model of the cylindrical magnet [17]. The measurement noise parameter of the Kalman filter corresponds to the standard deviation of the Hall sensors’ noise and amounts to 50 µT. The second crucial parameter, the process noise standard deviation, is derived from the expected movement speed of the magnet and set to p = 100 µm. 

## 4. Results and Discussion

A position detection along one axis is experimentally demonstrated and compared to theory. Subsequently, an integrable magnetic position system with three degrees of freedom is evaluated. 

### 4.1. One-Dimensional (1D) Measurement

A calibration curve of B_z_(h) is experimentally determined by retracting the magnet in steps of 10 µm up to h = 1000 µm. At each position 100 data points are recorded and arithmetically averaged. The resulting relation B_z_(h) is fitted with a fourth-degree polynomial function (least squares, R² = 99.98%) in the range of h = 0–1000 µm. After the magnet is again brought into contact with the surface of the sensor substrate, a second B_z_(h) measurement is performed. This time, the magnet is retracted in steps of 25 µm, with 100 data points acquired at each position. Using the reference function from the first run, the B_z_ data are converted to the corresponding displacement ∆z.

**Figure 7:**
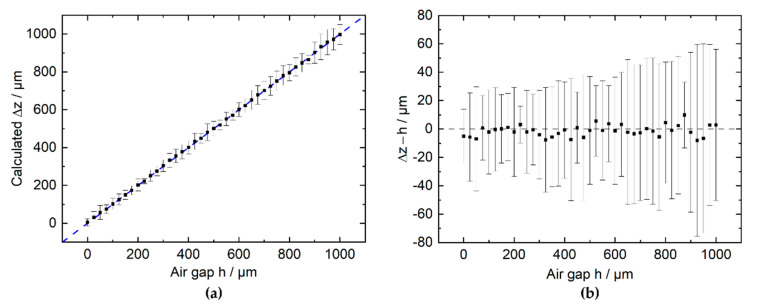
(**a**) ∆z calculated from the previously fitted calibration curve plotted with respect to the air gap h between the magnet and the sensor package surface. (**b**) Difference between calculated ∆z and actual air gap h. Each point represents a mean of 100 measurements of B_z_, with the bars indicating the 1σ interval of the 100 individually acquired measurements.

Figure 7a displays the calculated displacement ∆z with respect to the air gap h set by the manipulator. The difference of both positions ∆z-h is shown in Figure 7b. 

Upon averaging 100 measurements per position, the mean deviation within the range from 0 to 1000 µm is (−1.3 ± 4) µm with the maximum deviation of 9.8 µm. At a 10-fold higher bandwidth, i.e., an average of 10 measurements per position, the mean difference ∆z−h in the range from 0 to 1000 µm is (−1.1 ± 15) µm. 

**Figure 8:**
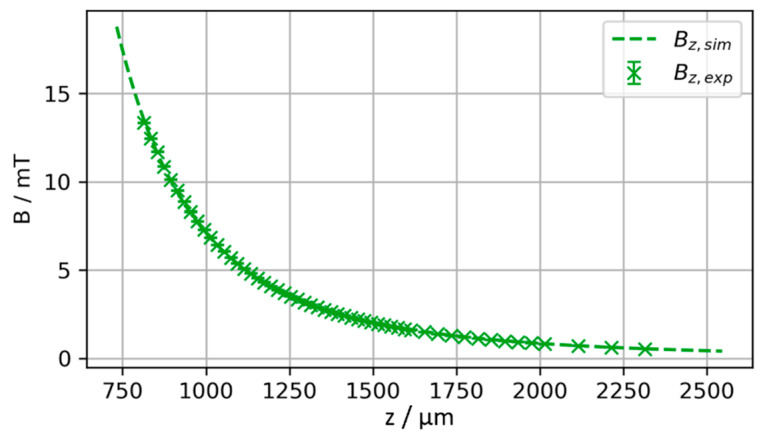
Result of fit of simulated versus experimentally measured magnetic field with z_0_ and M as degrees of freedom. The best fit is achieved for z_0_ = 814 µm, M = 357 mT/µ_0_.

To validate the experimental measurements, they are compared against the theoretically expected magnetic field obtained from numerical simulations by integrating the Biot–Savart law according to equation (1). [17,18] The starting position of the magnet z_0_ (including L/2 and t_Si_, refer to Figure 2) and the magnetization M are treated as degrees of freedom. This is necessary to handle the uncertainty in absolute positioning resulting from the sensor pixel cell position and the total magnetic moment of the permanent magnet. The air gap h measured in the experiment and the z position of the magnet used in the theoretical calculations are related by z = z_0_ + h. z_0_ and M are derived from fitting the experimental measurement to the theoretical magnetic field by minimizing the sum of squared residuals. The magnetization is assumed to be homogeneous and z-oriented, i.e., $\vec{M}=M_{z}\vec{e_{z}}$. Figure 8 compares the fit result to the experimental data. For z_0_ = 814 µm and M = 357 mT/µ_0_ a very good agreement between theoretically expected and measured magnetic flux density is achieved as shown in Figure 8. The resulting values for z_0_ and M are in the experimentally expected range, considering the geometry as illustrated in Figure 2 and the VSM data given in Figure 4.

**Figure 9:**
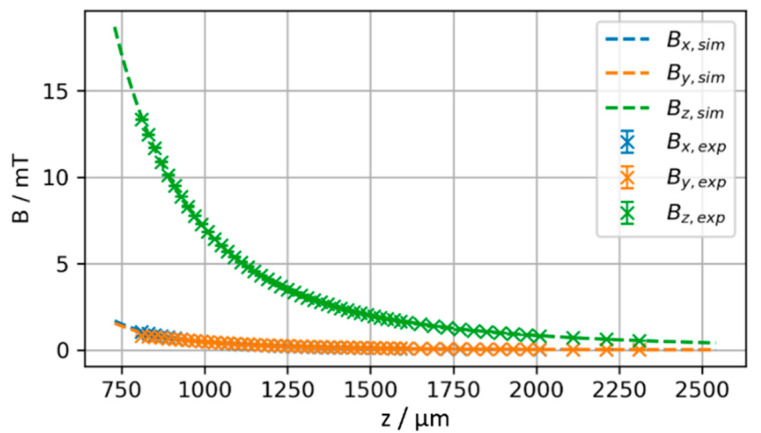
Fit of simulated versus experimentally measured magnetic field with x_0_, y_0_, z_0_ and M as degrees of freedom yielding x_0_ = 42 µm, y_0_ = 39 µm, z_0_ = 810 µm, M = 357 mT/µ_0_.

In the case of a perfect axial alignment of the sensor and the magnetization axis, both B_x_ and B_y_ are zero. Allowing an initial misalignment of x_0_ and y_0_ explains non-zero B_x_ and B_y_, as observed in the experiment. Adding x_0_ and y_0_ as additional degrees of freedom and B_x_ and B_y_ as fit targets, the fit converges to the solution shown in Figure 9. The resulting parameters x_0_ = 42 µm, y_0_ = 39 µm, z_0_ = 810 µm, M = 357 mT/µ_0_ are in close agreement to the above fit, but additionally indicate a misalignment of 57 µm in radial direction.

The above comparison shows that the experimental observations agree well with a model based on the theoretically expected magnetic field of a cylindrical permanent magnet. While a small lateral misalignment cannot be avoided in the chosen experimental setup, the transversal position detection exhibits only a low sensitivity regarding such a misalignment; the calculations indicate that a lateral misalignment of 57 µm leads to a difference in transversal position detection of 4 µm. For MEMS devices, lateral alignment can be further constrained in device design by allowing only one transverse degree of freedom in the moving structures. In manufacturing, precise axis-accurate assembly on wafer level can be achieved using established BEOL processes. Another approach to overcoming alignment problems and enabling further applications is the estimation of three degrees of freedom, as discussed in the following section.

### 4.2. Three-Dimensional (3D) Measurements

Having verified the magnetic model of the cylindrical magnet and the measurement accuracy along the z-axis, the position measurement can be extended to multiple degrees of freedom. 

**Figure 10:**
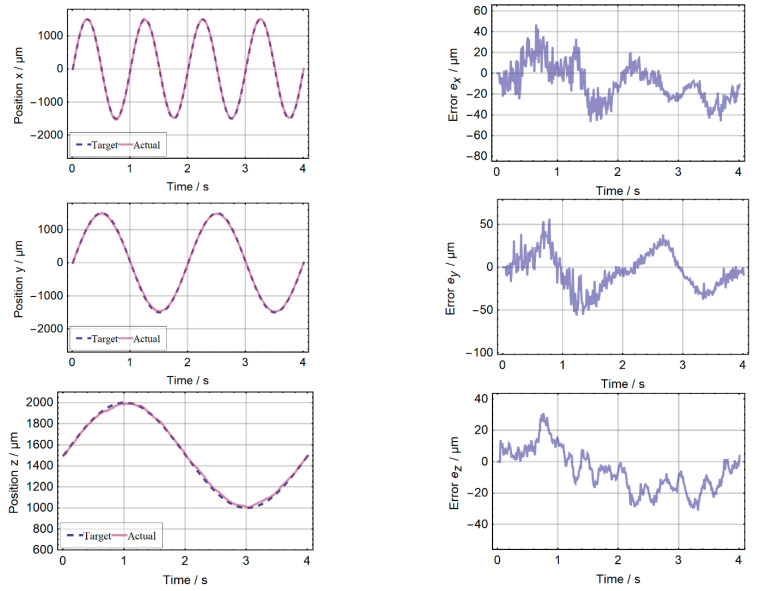
Result of the 3D position measurement. Right: target and estimated (actual) values for the position components x, y and z. Along the trajectory x and y vary within ±1.5 mm, z within ±0.5 mm. Left: the residual error for the individual components staying below 51 µm for all axes.

The position is estimated by the unscented Kalman filter as described in [8] and compared to the steering value of the 6D manipulator. To align the magnet and the platform, the latter is moved to the position at which the estimation for x and y approaches zero. This results in error curves that fluctuate around zero. A preceding 1D measurement with only one 3D Hall sensor (see Section 4.1) was used to derive the magnetization M = 239 mT/µ_0_. Figure 10 shows the results of the position estimation based on the Hall sensor measurements performed with the setup presented in Section 3.3. The remaining error depends on the distance of the magnet to the IC. For the whole trajectory, the position error of every axis is below 51 µm. If this position measurement system were to be used in a human interface device (e.g., joystick [7] or trackpoint) an effective number of bits (ENOB) of more than 4 bits in all three axes could be achieved. For such an application only a single sensor IC and the embedded magnet are necessary. Since no moving mechanical parts are required for electrical connections, such a system is very robust and durable. The calculation performed by the external microcontroller takes 2 ms. This corresponds to the measurement time of the Hall sensor and allows real-time operation.

## 5. Conclusions and Outlook

A fully BEOL integrable magnetic position detection was demonstrated by the combination of NdFeB micromagnets integrated on substrate level and CMOS Hall sensors. At a distance of up to 1500 µm, the system achieves precisions in the order of 1 µm to 10 µm, depending on bandwidth and distance. In 3D position detection, real-time operation with errors below 51 µm per axis was demonstrated. The system can be highly miniaturized and customized by application-specific arrangements such as arrays of Hall sensors and integrated magnets. 

Future work will include the compensation of external magnetic fields and temperature drift. Due to the symmetry and the small size of the utilized cylindrical magnet, the observability condition for six mechanical degrees of freedom was not fulfilled. In our future work, an optimized arrangement of multiple integrated micromagnets should enable complete estimation of position and orientation in space (6D position detection). Post-processing will be shifted to the sensor IC by integrating a RISC-V core. 

## 6. Patents

The wafer-level process for the creation of integrated micromagnets from powder by ALD solidification is patented under EP 2670880 B1, US 9221217 B2 and JP 6141197 B2. The integrated positioning system for microsystems based on integrated micromagnets is patent pending under DE 102019212091 A1 and WO 2021/028345 A2. The localization method and assembly are patent pending under DE 102015203686 A1.

## Supplementary Materials

The following are available online at https://www.mdpi.com/article/10.3390/mi13020235/s1, Video S1: animated 3D trajectory.
